# Impact of left ventricular end-diastolic diameter size within 24 hours of hospital admission on outcome events in patients with ST-elevation myocardial infarction

**DOI:** 10.7717/peerj.21108

**Published:** 2026-04-20

**Authors:** Lingling Zhang, Zhican Liu, Li Zhou, Li Peng, Yan Luo, Juan Chen, Jianping Zeng, Xianghong Zhou, Mingxing Wu, Yong Liang, MingYan Jiang

**Affiliations:** 1 Medical Department, Xiangtan Central Hospital, The Affiliated Hospital of Hunan University, Xiangtan, China; 2 Department of Pulmonary and Critical Care Medicine, Xiangtan Central Hospital, The Affiliated Hospital of Hunan University, Xiangtan, China; 3 Echocardiography Department, Xiangtan Central Hospital, The Affiliated Hospital of Hunan University, Xiangtan, China; 4 Department of Oncology, Xiangtan Central Hospital, The Affiliated Hospital of Hunan University, Xiangtan, China; 5 Department of Cardiology, Xiangtan Central Hospital, The Affiliated Hospital of Hunan University, Xiangtan, China; 6 Xiangtan Central Hospital, The Affiliated Hospital of Hunan University, Department of Scientific Research, Xiangtan, China

**Keywords:** ST-elevation myocardial infarction (STEMI), Left ventricular end-diastolic diameter (LVEDD), Percutaneous coronary intervention (PCI), Risk factors, Prognosis, Composite endpoint events

## Abstract

**Background:**

The prognosis of patients with ST-elevation myocardial infarction (STEMI) is closely related to the left ventricular end-diastolic diameter (LVEDD) size. However, the associations between LVEDD, pre-admission risk factors, admission biochemical markers, and medication use warrant further investigation. The prognostic impact of LVEDD evaluation within 24 hours of admission in STEMI patients has not been extensively studied.

**Methods:**

We analyzed the association between LVEDD measurements within 24 hours of admission and a composite endpoint of cardiovascular events and mortality in 664 STEMI patients undergoing percutaneous coronary intervention (PCI). Patients were categorized into three groups based on LVEDD size. Multiple regression models examined the relationship among pre-admission factors, biochemical markers, medication use, and LVEDD.

**Results:**

Composite endpoint events occurred in 249 patients. A larger admission LVEDD was associated with a higher risk of endpoint events (hazard ratio (HR) 1.032; 95% confidence interval (CI) [1.004–1.061]; *P* = 0.027), especially when LVEDD exceeded 47 mm (HR 1.605, 95% CI [1.185–2.174], *P* = 0.002) and 54 mm (HR 1.647, 95% CI [1.027–2.643], *P* = 0.039). Multivariate regression identified independent factors influencing composite endpoint events and LVEDD, including Killip classification ≥2, obesity, use of vasoactive drugs, pre-admission history of cardiomyopathy, NT-proBNP, uric acid, albumin, and the use of spironolactone, diuretics, and digoxin (All *P* values < 0.05).

**Conclusion:**

LVEDD measurement within 24 hours of admission is crucial for predicting composite endpoint events in STEMI patients. An elevated risk is observed in patients with LVEDD greater than 47 mm, which is further amplified when LVEDD surpasses 54 mm. Identifying independent factors influencing LVEDD and clinical outcomes provides valuable insights for clinical management.

## Introduction

ST-segment elevation myocardial infarction (STEMI) is recognized as one of the principal causes of mortality and morbidity worldwide (Thomas et al., 2018),  resulting from acute myocardial ischemia and consequent necrosis (Ibanez et al., 2018). Following acute myocardial infarction (AMI), the heart often undergoes left ventricular remodeling, which may include an increase in the left ventricular end-diastolic diameter (LVEDD). Such enlargement represents a critical risk factor for heart failure and death (Pfeffer & Braunwald, 1990; St John Sutton et al., 1994; Solomon et al., 2005). During myocardial ischemia, the death of myocardial cells leads to the release of intracellular substances, such as cardiac troponin (Thygesen et al., 2018). These substances can induce inflammatory reactions in myocardial and endothelial cells, leading to reduced contractile function and impaired diastolic function (Frangogiannis, 2012).

In addition to the myocardial ischemia and necrosis caused by STEMI, other factors can contribute to an increased LVEDD. Conditions such as hypertension (Whelton et al., 2018), valvular disease (Nkomo et al., 2006), cardiomyopathy (Maron et al., 2006), and myocarditis (Cooper, 2009) can all result in left ventricular enlargement. Specific lifestyle, genetic, and environmental factors may also increase LVEDD (Levy et al., 1990). For example, conditions such as diabetes, obesity, smoking, and hyperlipidemia can all increase the cardiac load, potentially leading to left ventricular dilation (Whelton et al., 2018). Furthermore, certain medications, such as diuretics, dobutamine hydrochloride, and digoxin, may also affect LVEDD (Yancy et al., 2017; Felker et al., 2020).

Previous research has established a correlation between increased LVEDD and poor prognosis in STEMI patients, including heart failure, death, and recurrent myocardial infarction (Van Loon et al., 2014; St John Sutton et al., 1994; Pfeffer et al., 1992). However, the impact of evaluating LVEDD within the first 24 h of hospital admission on the prognosis of STEMI patients has not been extensively studied. Early assessment of LVEDD in STEMI patients can aid in predicting prognosis and provide a foundation for early intervention and treatment planning. Consequently, this study aims to investigate the association between LVEDD measured within 24 h of admission and clinical outcomes in STEMI patients, thereby offering clinicians more targeted evaluation methods. Additionally, we examine pre-admission risk factors, biochemical markers, and medication usage associated with LVEDD to identify potential influencing factors LVEDD size and provide further insight for clinical interventions.

## Methods

### Patient screening and data collection

**Figure 1:**
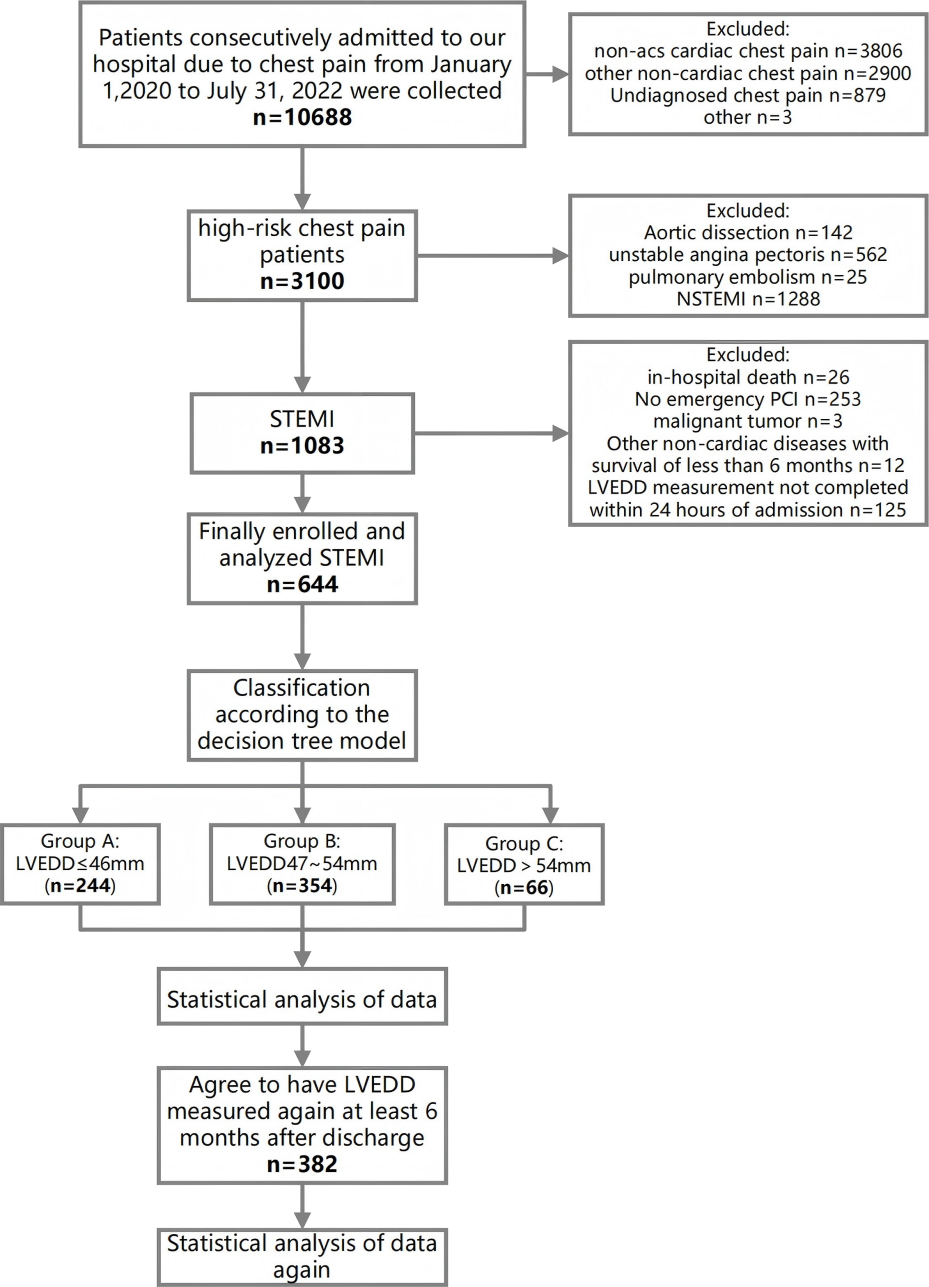
Flow diagram for participant screening, eligibility and analysis.

We conducted a retrospective study including 664 patients diagnosed with STEMI who underwent percutaneous coronary intervention (PCI) at our hospital between January 1, 2020, and July 31, 2022 (shown in Fig. 1). The median door-to-balloon time was 69.56 ± 26.90 minutes. We retrieved clinical data, including demographic, pre-admission medical history, admission biomarkers, medication usage, and PCI-related details, from the hospital’s electronic medical record system and the national chest pain platform.

### Follow-up and outcome events

The follow-up period for all study participants persisted until January 31, 2023, with a median follow-up duration of 531 days. A team comprising five experienced cardiovascular specialists and two nurses assessed patient outcomes through outpatient clinic visits, telephonic follow-ups, and community visits. For patients whose valid data could not be obtained through these standard methods, information was supplemented using the official data system of the Xiangtan Center for Disease Control and Prevention (Xiangtan CDC).

Outcome Events: The primary endpoint was a composite of cardiovascular events and mortality. Cardiovascular events were defined as non-fatal myocardial infarction, ischemic stroke, readmission for angina pectoris, or heart failure, bleeding, and repeat revascularization.

Inclusion criteria:

1. Patients diagnosed with a first episode of STEMI according to the established guidelines (Ibanez et al., 2018).
2. Completion of LVEDD measurement within 24 h of admission.
3. Patients undergoing emergency percutaneous coronary intervention (PCI).

Exclusion criteria:

1. Patients aged <18 years.
2. Patients lacking LVEDD measurement data.
3. Patients with missing essential data.
4. Patients who died during hospitalization.
5. Patients who did not undergo PCI.
6. Patients with malignant tumors or other non-cardiac conditions with a life expectancy of less than six months.

### LVEDD measurement

Transthoracic echocardiography was performed for all patients within 24 h of admission, specifically after the completion of the primary PCI procedure. This timing was selected to avoid any delay in reperfusion therapy. Using a decision tree model, patients were stratified into three groups based on LVEDD size: ≤ 46 mm, 47 mm–54 mm, and >54 mm. LVEDD was measured three times by two independent assessors, and the mean value was calculated. If the discrepancy between measurements exceeded 5%, an additional measurement was obtained, and the average was recalculated. Follow-up LVEDD measurements were obtained for 382 patients after a minimum of six months.

### Ethics and informed consent

This study was approved by the Ethics Committee of Xiangtan Central Hospital (Xiangtan, China) (Ethics Approval No. 2023-02-001) and adhered to the principles outlined in the Declaration of Helsinki. As a retrospective study that only collected clinical data without intervening in patient treatment, informed consent was waived.

### Statistical analysis

Continuous variables with a normal distribution were expressed as mean ± standard deviation (SD), while non-normally distributed variables were presented as median (interquartile range (IQR)). Categorical variables were expressed as frequencies (percentage). To compare clinical characteristics among the three groups, one-way analysis of variance (ANOVA) was used for normally distributed continuous variables, utilizing Tamhane’s T2 test for *post hoc* analysis when variances were unequal. The Kruskal-Wallis rank sum test was used for non-normally distributed continuous variables. Categorical variables were compared using the Chi-squared test or Fisher’s exact test, as appropriate.

Patients were stratified into three groups based on a decision tree model: ≤46 mm, 47–54 mm, and >54 mm. Survival analysis was performed using Kaplan–Meier curves, with the log-rank test used to evaluate differences in event rates among the LVEDD groups. Cox proportional hazards models were used to analyze the relationship between LVEDD and composite endpoint events and to assess the impact of covariates, with hazard ratios (HRs) and 95% confidence intervals (CIs) calculated. Additionally, a multiple linear regression model was used to identify independent factors associated with LVEDD size (including pre-admission factors, biochemical markers and medication use), adjusting for potential confounders such as age and gender.

A two-sided *P*-value < 0.05 was considered statistically significant. All statistical analyses were performed using R software version 4.2.0 (http://www.R-project.org) and EmpowerStats software (http://www.empowerstats.com, X&Y Solutions, Inc. Boston, MA).

## Results

### Clinical profiles

**Figure 2:**
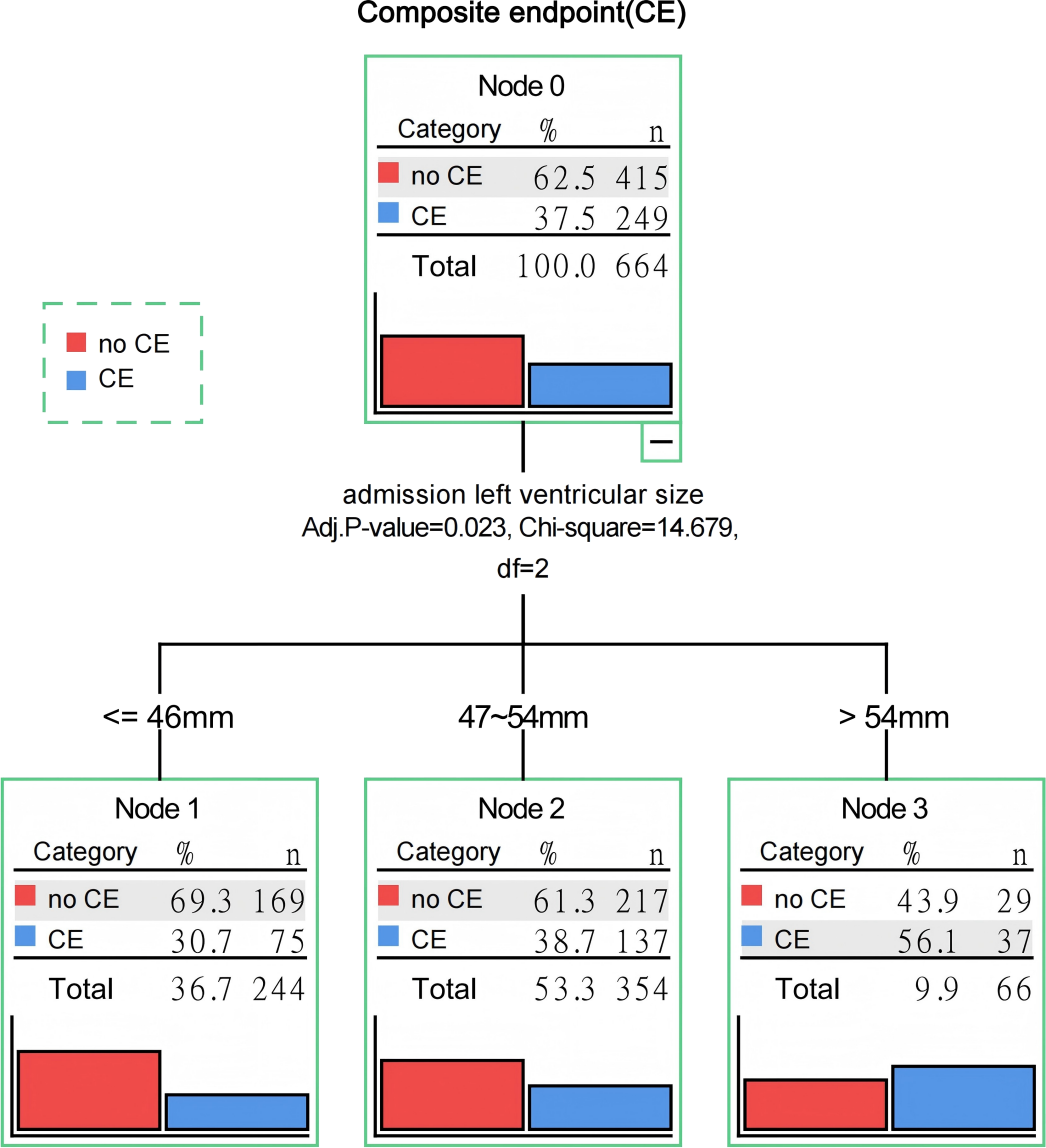
Potential cutoff value of admission left ventricular size associated with composite endpoint defined by Chi-square Automatic Interaction Detector (CHAID) decision trees.

Based on the decision tree model (Fig. 2) utilizing endpoint events and admission LVEDD, the 664 STEMI patients undergoing PCI were divided into three groups: 244 patients (36.75%) with LVEDD ≤ 46 mm (Group A), 354 patients (53.31%) with LVEDD ranging from 47–54 mm (Group B), and 66 patients (9.94%) with LVEDD > 54 mm (Group C).

As shown in Table 1, compared to Group A, Groups B and Group C exhibited significantly higher proportions of female patients, smokers, and comorbidities including obesity, heart valve disease, cardiomyopathy and renal insufficiency. Additionally, the prevalence of Killip classification ≥2 and the use of ARNI, diuretics, spironolactone, vasoactive drugs, positive inotropic drugs, and digoxin were significantly higher in these groups. Levels of admission heart rate, NT-proBNP, hemoglobin, uric acid and creatinine were also significantly elevated in Groups B and C. Conversely, the prevalence of hypertension and anemia, as well as levels of pulse pressure, high-density lipoprotein, albumin, and left ventricular ejection fraction (LVEF), were significantly lower in Groups B and C (all *p* < 0.05).

**Table 1:** Baseline characteristics by admission LVEDD size group.

	**Admission** ** LVEDD** ** size**		
**Variables**	≤46 mm (*n* = 244, Group A)	47∼54 mm (*n* = 354, Group B)	>54 mm (*n* = 66, Group C)
**Demographics**			
Male, N (%)	147 (60.250%)	308 (87.010%)^*^	60 (90.911%)^*^
Age, years	63.020 ± 12.491	61.691 ± 12.041	63.181 ± 10.682
Smoker, N (%)	113 (46.312%)	231 (65.253%)^*^	34 (51.520%)
Drinker, N (%)	33 (13.522%)	54 (15.252%)	8 (12.121%)
Obesity, N (%)	55 (22.542%)	114 (32.202%)^*^	15 (22.731%)
Systolic blood pressure, mmHg	129.381 ± 23.771	126.341 ± 24.361	123.420 ± 28.580
pulse pressure, mmHg	50.341 ± 14.710	48.891 ± 15.160	45.481 ± 13.820^*^
Admission heart rate, bpm	80.890 ± 15.111	78.832 ± 16.422	89.521 ± 21.341^*^^a^
**Medical history, N (%)**			
Hyperlipidemia	93 (38.111%)	130 (36.721%)	30 (45.451%)
Hypertension	154 (63.111%)	188 (53.110%)^*^	40 (60.610%)
Atrial fibrillation	24 (9.840%)	20 (5.650%)	8 (12.120%)
Diabetes mellitus	79 (32.380%)	90 (25.420%)	17 (25.760%)
Hyperthyroidism	5 (2.050%)	9 (2.541%)	1 (1.521%)
Stroke	31 (12.700%)	42 (11.860%)	13 (19.701%)
Heart valve disease	38 (15.570%)	45 (12.711%)	20 (30.300%)^*^^a^
Cardiomyopathy	5 (2.052%)	15 (4.242%)^*^	6 (9.092%)
COPD	30 (12.300%)	46 (12.990%)	11 (16.670%)
Renal insufficiency	36 (14.750%)	44 (12.430%)	18 (27.270%)^*^^a^
Anemia	55 (22.540%)	46 (12.991%)^*^	14 (21.211%)
Killip classification≥2	89 (36.481%)	128 (36.161%)	45 (68.182%)^*^^a^
**Serology**			
NT-proBNP, pg/ml	1,515.320 ± 3,118.471	1,420.631 ± 2,920.950	4,182.671 ± 6,367.531^*^^,^^a^
TnT, ng/mL	4.790 ± 3.551	5.030 ± 3.520	5.340 ± 3.940
Hemoglobin,g/L	132.180 ± 18.550	137.241 ± 16.961^*^	135.111 ± 15.011
Glycosylated hemoglobin,%	6.341 ± 1.541	6.151 ± 1.281	6.421 ± 1.461
Thyrotropin, uIU/ml	1.821 ± 1.461	1.481 ± 1.071^*^	1.981 ± 2.181^a^
Uric acid, µmol/L	323.271 ± 94.791	343.971 ± 94.931^*^	391.731 ± 110.771^*^^,^^a^
Creatinine, µmol/L	80.641 ± 34.341	85.531 ± 62.401	106.681 ± 143.781^*^
Total cholesterol,mmol/L	4.491 ± 0.991	4.361 ± 1.081	4.500 ± 1.201
Triglyceride, mmol/L	2.211 ± 2.691	2.031 ± 1.941	1.560 ± 0.811
Low density lipoprotein,mmol/L	2.921 ± 0.841	2.872 ± 0.930	3.090 ± 1.080
High density lipoprotein,mmol/L	1.030 ± 0.310	0.970 ± 0.241^*^	1.030 ± 0.261
Albumin,g/L	40.381 ± 3.980	40.571 ± 4.300	39.061 ± 3.721^a^
**Echocardiography**			
Aortic size,mm	32.811 ± 3.350	33.850 ± 3.240^*^	34.530 ± 3.780^*^
Left atrial size,mm	33.270 ± 3.510	35.790 ± 3.550^*^	40.380 ± 5.990^*^^,^^a^
LVEDD size,mm	44.060 ± 1.970	49.700 ± 2.170^*^	59.080 ± 4.020^*^^,^^a^
Right atrial size,mm	35.080 ± 3.640	36.490 ± 3.040^*^	38.380 ± 4.460^*^^,^^a^
Right ventricular size,mm	30.890 ± 3.920	32.250 ± 3.100^*^	33.442 ± 3.880^*^^,^^a^
LVEF, %	53.860 ± 7.570	50.930 ± 7.550^*^	39.120 ± 9.990^*^^,^^a^
**Treatment, N (%)**			
Beta-blocker	224 (91.800%)	305 (86.160%)	61 (92.420%)
ACEI	87 (35.660%)	119 (33.620%)	19 (28.790%)
ARB	84 (34.430%)	122 (34.460%)	17 (25.760%)
ARNI	87 (35.660%)	142 (40.110%)	40 (60.610%)^*^^,^^a^
SGLT2i	21 (8.610%)	26 (7.340%)	7 (10.611%)
Diuretics	130 (53.280%)	183 (51.690%)	56 (84.850%)^*^^,^^a^
Spironolactone	41 (16.800%)	79 (22.320%)	32 (48.480%)^*^^,^^a^
Statins	244 (100.000%)	348 (98.310%)	66 (100.000%)
Antiplatelet Drugs	244 (100.000%)	349 (98.590%)	66 (100.000%)
Anticoagulant drugs	46 (18.850%)	70 (19.770%)	19 (28.790%)
Vasoactive drugs	31 (12.701%)	43 (12.151%)	18 (27.271%)^*^^,^^a^
Positive inotropic drugs	8 (3.281%)	17 (4.800%)	11 (16.671%)^*^^,^^a^
Digoxin	3 (1.231%)	5 (1.411%)	6 (9.090%)^*^^,^^a^
Vasodilators	205 (84.020%)	284 (80.231%)	52 (78.792%)
**Outcome event, N (%)**			
Composite endpoint	75 (30.740%)	137 (38.700%)	37 (56.062%)^*^^,^^a^

**Notes.**

The population was classified according by admission left ventricular size. Values for continuous variables are given as means ± SD. Group A: Admission left ventricular size ≤46 mm; Group B: Admission left ventricular size 47∼54 mm; Group C: Admission left ventricular size >54 mm.

* *P* < 0.050 *vs.* Group A.

a *P* < 0.050 *vs.* Group B.

Abbreviations COPD chronic obstructive pulmoriary disease NT-proBNP N-terminal pro-B type natriureti peptide TnT Troponin T LVEF left ventricular ejection fraction ACEI angiotensin-converting enzyme inhibitors ARB angiotensin receptor blockers ARNI angiotensin receptor - enkephalase inhibitors SGLT2i sodium-dependent glucose transporters 2 inhibitors LVEDD left ventricular end-diastolic diameter

### Clinical outcome

**Figure 3:**
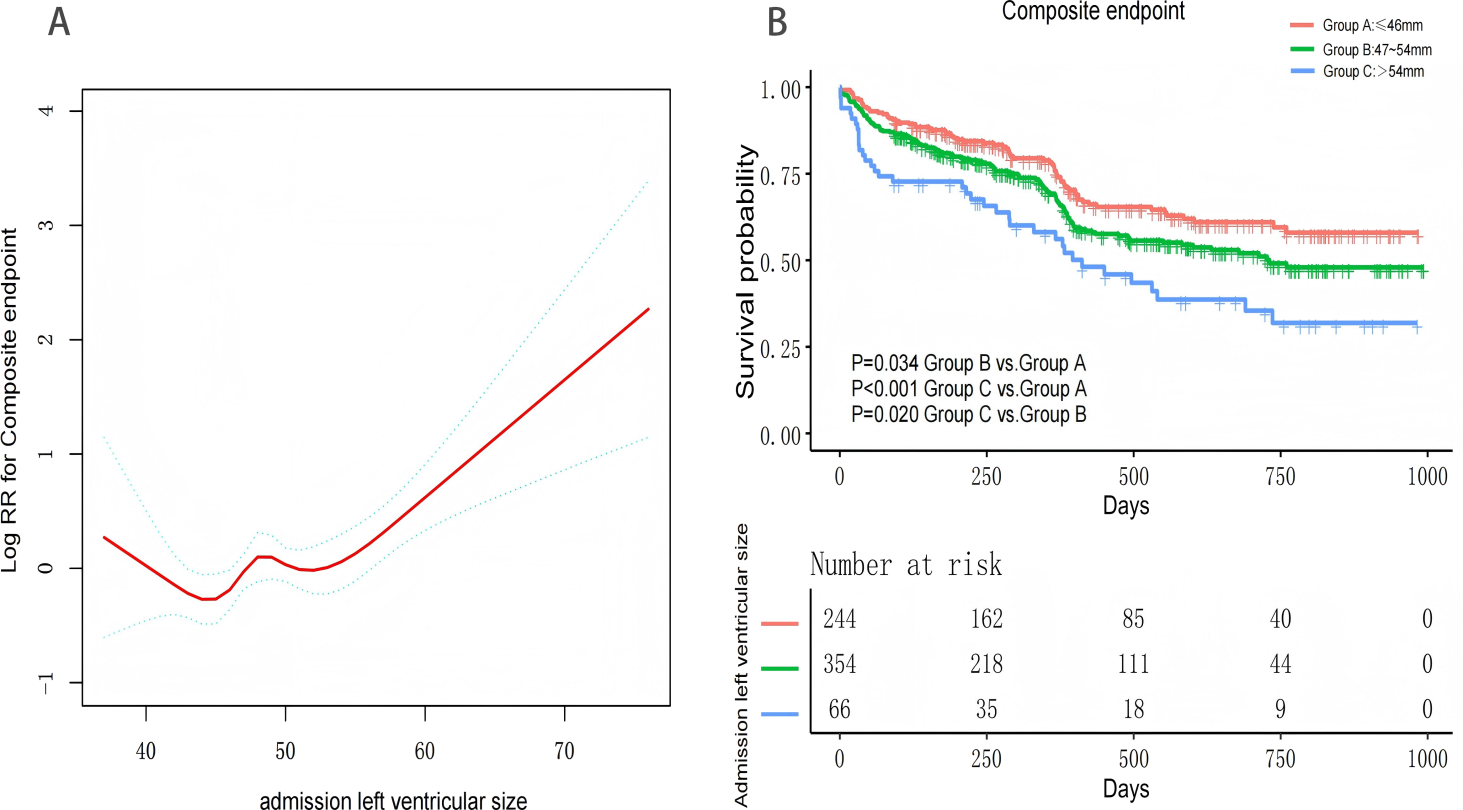
Trend graph of LVEDD size and composite endpoint. (A) Curve fitting of LVEDD size to the composite endpoint (B) Cumulative incidence of the composite endpoint across groups.

Table 2 presents the association between admission LVEDD (analyzed as both a continuous and categorical variable) and composite endpoint events. In the unadjusted model (Model I), the risk of composite endpoint events increased with increasing admission LVEDD as a continuous variable (hazard ratio (HR) 1.044; 95% confidence interval (CI) [1.019–1.070]; *P* < 0.001), as illustrated in Fig. 3A. When analyzed as a categorical variable, patients in Group B (HR 1.356; 95% CI [1.023–1.797]; *P* = 0.034) and Group C (HR 2.129; 95% CI [1.436–3.157]; *P* < 0.001) exhibited a significantly higher risk of composite endpoints compared to Group A (Fig. 3B).

**Table 2:** Effect of admission LVEDD size on clinical outcome.

**Variables**	**Hazard ratio** ** (95% CI)**	***P*-value**
Model I		
Admission LVEDD size	1.044 (1.019, 1.070)	**<0**.**001**
Admission LVEDD size grouping		
≤46 mm	ref.	
47∼54 mm	1.356 (1.023, 1.797)	**0**.**034**
>54 mm	2.129 (1.436, 3.157)	**<0**.**001**
Model VII		
Admission LVEDD size	1.032 (1.004, 1.061)	**0**.**027**
Admission LVEDD size grouping		
≤46 mm	ref.	
47∼54 mm	1.605 (1.185, 2.174)	**0**.**002**
>54 mm	1.647 (1.027, 2.643)	**0**.**039**

**Notes.**

Hazard ratios from Cox proportional hazards regressions. Bold represent significant values (*p* < 0.05).

Model I adjust for: None.

Model VII adjust for: Age; Smoker; Drinker; Obesity; Hyperlipidemia; Hypertension; Atrial fibrillation; Diabetes mellitus; Hyperthyroidism; Stroke; Heart valve disease; Cardiomyopathy; Chronic obstructive pulmonary disease; Renal insufficiency; Anemia; Killip classification; N-terminal pro-B type natriureti peptide; Troponin T; High density lipoprotein; Creatinine; Albumin; Angiotensin receptor neprilysin Inhibitor; Diuretics; Spironolactone; Vasoactive drugs; Digoxin.

Abbreviations CI confidence interval LVEDD left ventricular end-diastolic diameter

After adjustment for all confounding factors in Model VII, the risk of composite endpoints increased with an increase in admission LVEDD as a continuous variable (HR 1.032; 95% CI [1.004–1.061]; *P* = 0.027). Consistent results were observed in other models, where the risk of composite endpoints all increased with LVEDD as a continuous variable (Table S1). When admission LVEDD was analyzed as a categorical variable, patients in Group B (HR 1.605; 95% CI [1.185–2.174]; *P* = 0.002) and Group C (HR 1.647; 95% CI [1.027–2.643]; *P* = 0.039) maintained a significantly higher risk of composite endpoints compared to Group A (see Table 3 for details).

**Table 3:** Relationship between LVEDD size re-evaluated and composite endpoint after hospital discharge.

**Variables**	**Model** I		**Model** II		**Model** III	
	**Hazard ratio (95% CI)**	***P*-value**	**Hazard ratio (95% CI)**	***P*-value**	**Hazard ratio (95% CI)**	***P*-value**
Follow-up LVEDD size	1.058 (1.032, 1.084)	<0.001	1.059 (1.032, 1.086)	<0.001	1.051 (1.019, 1.083)	0.001
LVEDD size difference	1.061 (1.024, 1.100)	0.001	1.061 (1.023, 1.101)	0.001	1.045 (1.003, 1.088)	0.037
Rate of change in LVEDD size	1.028 (1.011, 1.046)	0.001	1.028 (1.011, 1.046)	0.002	1.021 (1.001, 1.041)	0.035

**Notes.**

Hazard ratios from Cox proportional hazards regressions. Bold represent significant values (*p* < 0.05).

Model I adjust for: None.

Model II adjust for: Gender; Age.

Model III adjust for: Age; Smoker; Drinker; Obesity; Hyperlipidemia; Hypertension; Atrial fibrillation; Diabetes mellitus; Hyperthyroidism; Stroke; Heart valve disease; Cardiomyopathy; Chronic obstructive pulmonary disease; Renal insufficiency; Anemia; Killip classification; N-terminal pro-B type natriureti peptide; Troponin T; High density lipoprotein; Creatinine; Albumin; Angiotensin receptor neprilysin Inhibitor; Diuretics; Spironolactone; Vasoactive drugs; Digoxin.

Abbreviations CI confidence interval LVEDD left ventricular end-diastolic diameter

We also conducted separate analyses for mortality and cardiovascular adverse events. LVEDD as a continuous variable was positively associated with the risk of mortality (HR = 1.085, CI [1.030–1.144], *P* = 0.002), However, no independent association was observed with cardiovascular adverse events (HR = 1.022, CI [0.992–1.054]; *P* = 0.157) (Tables S3 and S4).

After a minimum follow-up period of six months, 382 of the 664 enrolled patients completed a follow-up LVEDD measurement; their baseline characteristics are presented in Table S2 . After adjustment for all confounding factors, an increase in follow-up LVEDD was associated with an elevated risk of composite endpoint occurrence (HR 1.051; 95% CI [1.019–1.083]; *P* = 0.001). Moreover, both the absolute difference (HR 1.045; 95% CI [1.003–1.088]; *P* = 0.037) and rate of change (HR 1.021; 95% CI [1.001–1.041]; *P* = 0.035) between the two LVEDD measurements were associated with an increased risk of composite endpoint (Table 4).

**Table 4:** Independent risk factors that impact LVEDD size upon hospital admission.

	**Univariable**		**Multivariable**	
**Variables**	**β(95% CI)**	***P*-value**	**β (95% CI)**	***P*-value**
**Demographics**				
Male	3.375 (2.506, 4.244)	**<0**.**001**	4.0528 (2.955, 5.150)	**<0**.**001**
Age	–0.005 (–0.036, 0.027)	0.780		
Smoker	0.709 (–0.053, 1.470)	0.069		
Drinker	–0.245 (–1.325, 0.835)	0.657		
**Past medical history**				
Obesity	0.462 (–0.382, 1.306)	0.284		
Hypertension	–0.461 (–1.226, 0.302)	0.237		
Hyperlipidemia	0.396 (–0.382, 1.174)	0.319		
Atrial fibrillation	–0.041 (–1.449, 1.366)	0.954		
Diabetes mellitus	–0.266 (–1.108, 0.576)	0.536		
Hyperthyroidism	–0.024 (–2.569, 2.520)	0.985		
Valvular heart disease	1.179 (0.138, 2.220)	**0**.**027**	0.589 (–0.408, 1.586)	0.247
Cardiomyopathy	2.903 (0.966, 4.840)	**0**.**003**	2.409 (0.598, 4.219)	**0**.**009**
COPD	0.192 (–0.929, 1.313)	0.737		
Renal insufficiency	1.369 (0.308, 2.430)	**0**.**012**	–0.793 (–1.938, 0.352)	0.175
Anaemia	–0.264 (–1.263, 0.735)	0.605		
**In-hospital examination results**				
Systolic blood pressure	–0.012 (–0.027, 0.004)	0.139		
Pulse pressure	–0.031 (–0.057, –0.006)	**0**.**015**	–0.021 (–0.061, 0.018)	0.283
Heart rate	0.036 (0.014, 0.059)	**0**.**002**	0.021 (–0.002, 0.043)	0.069
Hemoglobin	0.023 (0.001, 0.044)	**0**.**041**	0.012 (–0.016, 0.039)	0.397
NT-proBNP	0.0003 (0.0002, 0.0004)	**<0**.**001**	0.0003 (0.0002, 0.0005)	**<0**.**001**
TnT	0.041 (–0.065, 0.147)	0.444		
Glycosylated hemoglobin	0.013 (–0.257, 0.282)	0.928		
TSH	0.240 (–0.035, 0.514)	0.087		
Uric acid	0.012 (0.0080, 0.015)	**<0**.**001**	0.009 (0.005, 0.013)	**<0**.**001**
Cr	0.010 (0.005, 0.016)	**<0**.**001**	–0.004 (–0.011, 0.003)	0.217
TC	–0.140 (–0.497, 0.216)	0.441		
TG	–0.155 (–0.328, 0.018)	0.080		
LDL	0.175 (–0.239, 0.590)	0.407		
HDL	–1.516 (–2.924, –0.107)	**0**.**035**	–0.563 (–2.290, 1.165)	0.524
Albumin	–0.102 (–0.193, –0.011)	**0**.**028**	–0.104 (–0.202, –0.005)	**0**.**040**

**Notes.**

β values from multiple linear regression. Bold represent significant values (*p* < 0.050).

Abbreviations COPD Chronic obstructive pulmonary disease NT-proBNP N-terminal pro-B type natriureti peptide TnT Troponin T TSH Thyrotropin Cr Creatinine TC Total cholesterol TG Triglyceride LDL Low density lipoprotein HDL High density lipoprotein CI confidence interval LVEDD left ventricular end-diastolic diameter

### Independent risk factors affecting LVEDD size

**Figure 4:**
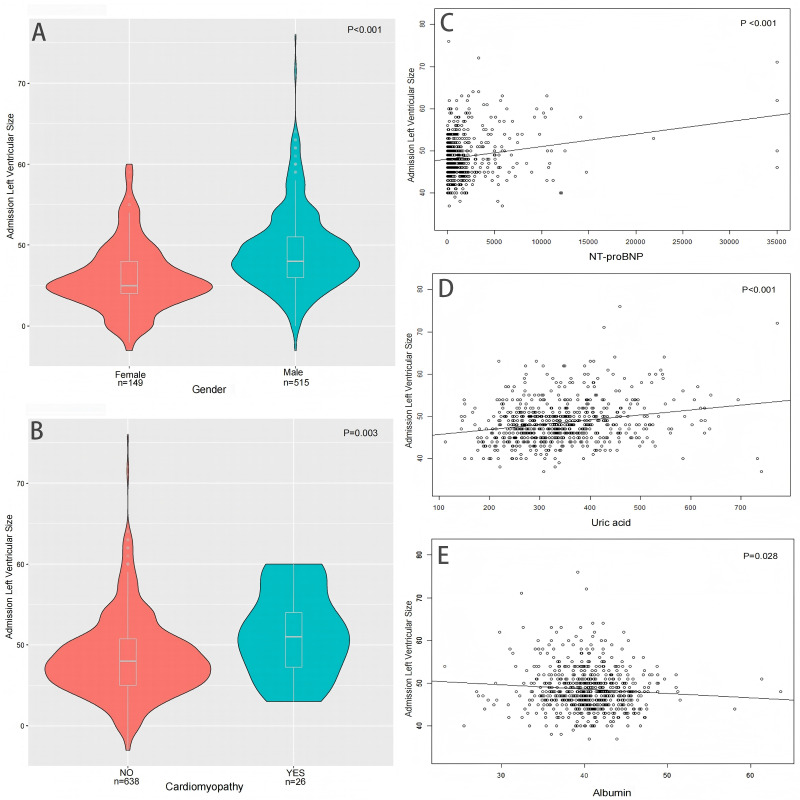
Factors affecting the size of LVEDD on admission. (A) Distribution of LVEDD size between men and women upon admission (B) Distribution of LVEDD size with or without cardiomyopathy upon admission (C) Relationship between NT-proBNP and LVEDD size upon admission (D) Relationship between uric acid and LVEDD size upon admission (E) Relationship between albumin and LVEDD size upon admission.

Table 5 presents the results of the univariate linear regression analysis, pre-admission factors (male gender, valvular heart disease, cardiomyopathy, renal insufficiency), and admission examination indicators (pulse pressure, heart rate, hemoglobin, NT-proBNP, uric acid, creatinine, high density lipoprotein, albumin), were found to be significantly correlated with admission LVEDD (all *P* < 0.05). Multivariable linear regression analysis revealed that male gender (β 4.0528, 95% CI [2.9552–5.1503], *P* < 0.001), pre-admission history of cardiomyopathy (β 2.4086, 95% CI [0.5982–4.2189], *P* = 0.009), admission NT-proBNP (β 0.0003, 95% CI [0.0002–0.0005], *P* < 0.001), uric acid (β 0.0087, 95% CI [0.0048–0.0126], *P* < 0.001), and albumin (β −0.1035, 95% CI [−0.2019–0.0051], *P* = 0.040) were independently associated with admission LVEDD (Fig. 4).

**Table 5:** Independent risk factors for follow-up LVEDD size.

**Variables**	**Univariable**		**Multivariable**	
	**β (95% CI)**	***P*-value**	**β (95% CI)**	***P*-value**
Male	3.603 (2.245, 4.961)	**<0**.**001**	3.6986 (2.437, 4.960)	**<0**.**001**
NT-proBNP	0.0005 (0.0004, 0.0007)	**<0**.**001**	0.0005 (0.0003, 0.0006)	**<0**.**001**
Spironolactone	3.257 (1.944, 4.571)	**<0**.**001**	1.715 (0.463, 2.968)	**0**.**008**
Diuretics	2.639 (1.496, 3.783)	**<0**.**001**	1.150 (0.060, 2.239)	**0**.**039**
Uric acid	0.012 (0.006, 0.018)	**<0**.**001**	0.007 (0.001, 0.0120)	**0**.**020**
Digoxin	8.129 (3.581, 12.676)	**<0**.**001**	4.383 (0.169, 8.597)	**0**.**042**
Albumin	–0.197 (–0.338, –0.056)	**0**.**006**	–0.144 (–0.275, –0.014)	**0**.**031**
SGLT2i	2.887 (0.737, 5.036)	**0**.**009**	1.382 (–0.548, 3.313)	0.161
Cardiomyopathy	2.873 (0.369, 5.377)	**0**.**025**	2.705 (0.532, 4.879)	**0**.**015**
Vasoactive drugs	1.723 (–0.034, 3.480)	0.055		
Beta-blocker	1.356 (–0.582, 3.293)	0.171		
Age	–0.015 (–0.065, 0.036)	0.570		

**Notes.**

β values from multiple linear regression. Bold represent significant values (*p* < 0.050).

Abbreviations NT-proBNP N-terminal pro-B type natriureti peptide SGLT2i sodium-dependent glucose transporters 2 inhibitors CI confidence interval LVEDD left ventricular end-diastolic diameter

**Figure 5:**
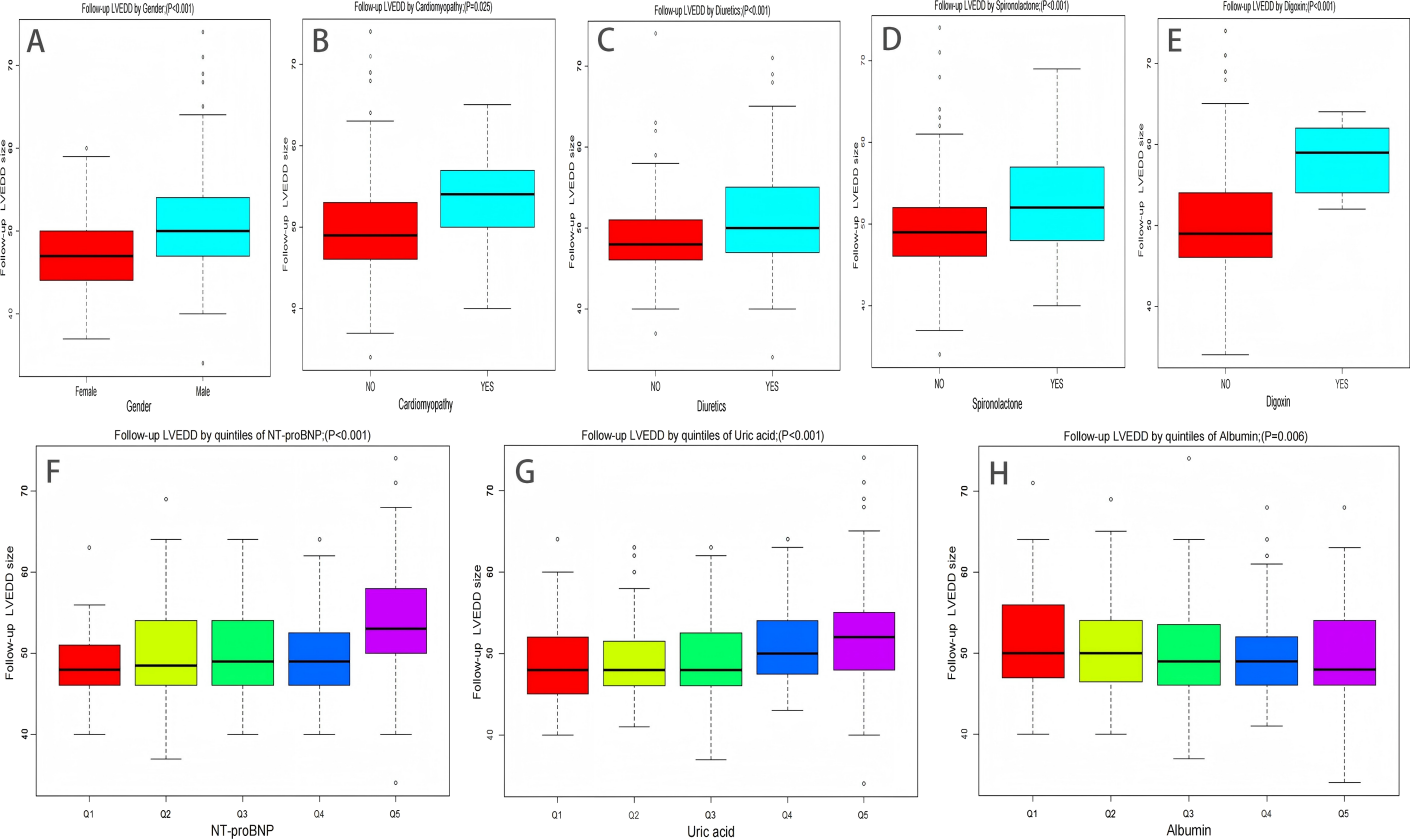
Factors affecting follow-up LVEDD size. (A) Difference in follow-up LVEDD size between men and women (B) Difference in follow-up LVEDD size with or without cardiomyopathy (C) Difference in follow-up LVEDD size with or without diuretics (D) Difference in follow-up LVEDD size with or without spironolactone (E) Difference in follow-up LVEDD size with or without digoxin (F) Relationship between NT-proBNP and follow-up LVEDD size (G) Relationship between uric acid and follow-up LVEDD size (H) Relationship between albumin and follow-up LVEDD size.

For the analysis of follow-up LVEDD, 382 patients with repeat measurement data were included. A new multivariable linear regression model was constructed, incorporating age, medication use, and the independent factors identified in Table 1 (Table 5). The results demonstrated that follow-up LVEDD increased with elevated NT-proBNP (β 0.0005, 95% CI [0.0003–0.0006], *P* < 0.001) and uric acid levels (β 0.0065, 95% CI [0.0010–0.0119], *P* = 0.020). Patients treated with spironolactone (β 1.7154, 95% CI [0.4630–2.9678], *P* = 0.008), diuretics (β 1.1495, 95% CI [0.0597–2.2392], *P* = 0.039), and digoxin (β 4.3829, 95% CI [0.1687–8.5972], *P* = 0.042) had significantly larger follow-up LVEDD values compared to those not receiving these medications. A previous history of cardiomyopathy (β 2.7054, 95% CI [0.5320–4.8789], *P* = 0.015) was identified as an independent risk factor for increased follow-up LVEDD. Conversely, albumin (β −0.1443, 95% CI [−0.2749–0.0137], *P* = 0.040) was identified as an independent protective factor against increased follow-up LVEDD (Fig. 5).

### Independent factors influencing outcome events

In the univariate analysis, NT-proBNP, use of vasoactive drugs, Killip classification ≥2, admission LVEDD, obesity, age, cardiomyopathy, heart valve disease, creatinine, digoxin, stroke, atrial fibrillation, and hypertension were all found to be significantly associated with composite endpoint events (all *P* < 0.05). After adjustment for all covariates, the use of vasoactive drugs (HR 1.6980; 95% CI [1.1721–2.4600]; *P* = 0.005), Killip classification ≥2 (HR 1.4595; 95% CI [1.0863–1.9610]; *P* = 0.012), and increased admission LVEDD (HR 1.0312; 95% CI [1.0030–1.0603]; *P* = 0.030) were identified as independent risk factors for composite endpoint events. Conversely, obesity (HR 0.5921; 95% CI [0.4247–0.8256]; *P* = 0.002) was identified as an independent protective factor (Table 6).

**Table 6:** Cox proportional hazards regression model analysis for risk of composite endpoint.

	**Univariable**			**Multivariable**	
**Variable**	**Hazard ratio (95% CI)**	**Wald**	***P*-value**	**Hazard ratio (95% CI)**	***P*-value**
NT-proBNP	1.000 (1.000, 1.000)	**21**.**09**	**<0**.**001**	1.000 (1.000, 1.000)	0.169
Vasoactive drugs	1.968 (1.449, 2.672)	**18**.**81**	**<0**.**001**	1.698 (1.172, 2.460)	**0**.**005**
Killip classification≥2	1.659 (1.294, 2.128)	**15**.**9**	**<0**.**001**	1.460 (1.086, 1.961)	**0**.**012**
LVEF	0.974 (0.960, 0.987)	**14**.**21**	**<0**.**001**	0.994 (0.973, 1.015)	0.560
Admission LVEDD size	1.044 (1.019, 1.070)	**12**.**1**	**<0**.**001**	1.031 (1.003, 1.060)	**0**.**030**
Obesity	0.583 (0.426, 0.799)	**11**.**25**	**<0**.**001**	0.592 (0.425, 0.826)	**0**.**002**
Age	1.018 (1.007, 1.029)	**11**.**05**	**<0**.**001**	1.010 (0.997, 1.024)	0.131
Cardiomyopathy	2.027 (1.283, 3.203)	**9**.**16**	**0**.**002**	1.430 (0.862, 2.372)	0.165
Heart valve disease	1.568 (1.153, 2.134)	**8**.**21**	**0**.**004**	1.292 (0.926, 1.802)	0.132
Creatinine	1.002 (1.000, 1.003)	**6**.**52**	**0**.**011**	1.001 (0.999, 1.003)	0.368
Digoxin	2.339 (1.155, 4.738)	**5**.**57**	**0**.**018**	1.270 (0.552, 2.923)	0.574
Stroke	1.497 (1.067, 2.100)	**5**.**45**	**0**.**020**	1.348 (0.941, 1.930)	0.103
Atrial fibrillation	1.616 (1.076, 2.426)	**5**.**35**	**0**.**021**	1.078 (0.682, 1.703)	0.749
Hypertension	1.302 (1.008, 1.681)	**4**.**08**	**0**.**043**	1.298 (0.988, 1.706)	0.061
Spironolactone	1.305 (0.977, 1.743)	3.24	0.072		
Anemia	1.309 (0.962, 1.782)	2.93	0.087		
ARNI	1.236 (0.954, 1.601)	2.56	0.110		
Albumin	0.9754 (0.9458, 1.0060)	2.5	0.114		
Diabetes mellitus	1.2265 (0.9364, 1.6064)	2.2	0.138		
Renal insufficiency	1.2751 (0.9232, 1.7611)	2.18	0.140		
Male	0.8120 (0.6120, 1.0775)	2.08	0.149		
Diuretics	1.1860 (0.9201, 1.5287)	1.73	0.188		
Smoker	0.8715 (0.6792, 1.1184)	1.17	0.280		
Hyperlipidemia	0.8771 (0.6772, 1.1359)	0.99	0.320		
Drinker	0.8390 (0.5788, 1.2162)	0.86	0.354		
COPD	0.8401 (0.5669, 1.2448)	0.75	0.385		
Hyperthyroidism	0.6504 (0.2421, 1.7476)	0.73	0.394		
High density lipoprotein	1.1888 (0.7595, 1.8606)	0.57	0.449		
TnT	1.0117 (0.9769, 1.0477)	0.42	0.515		

**Notes.**

Hazard ratios from Cox proportional hazards regressions. Bold represent significant values (*p* < 0.05).

Abbreviations COPD Chronic obstructive pulmonary disease NT-proBNP N-terminal pro-B type natriureti peptide TnT Troponin T ARNI angiotensin receptor - enkephalase inhibitors CI confidence interval LVEDD left ventricular end-diastolic diameter

## Discussion

**Figure 6:**
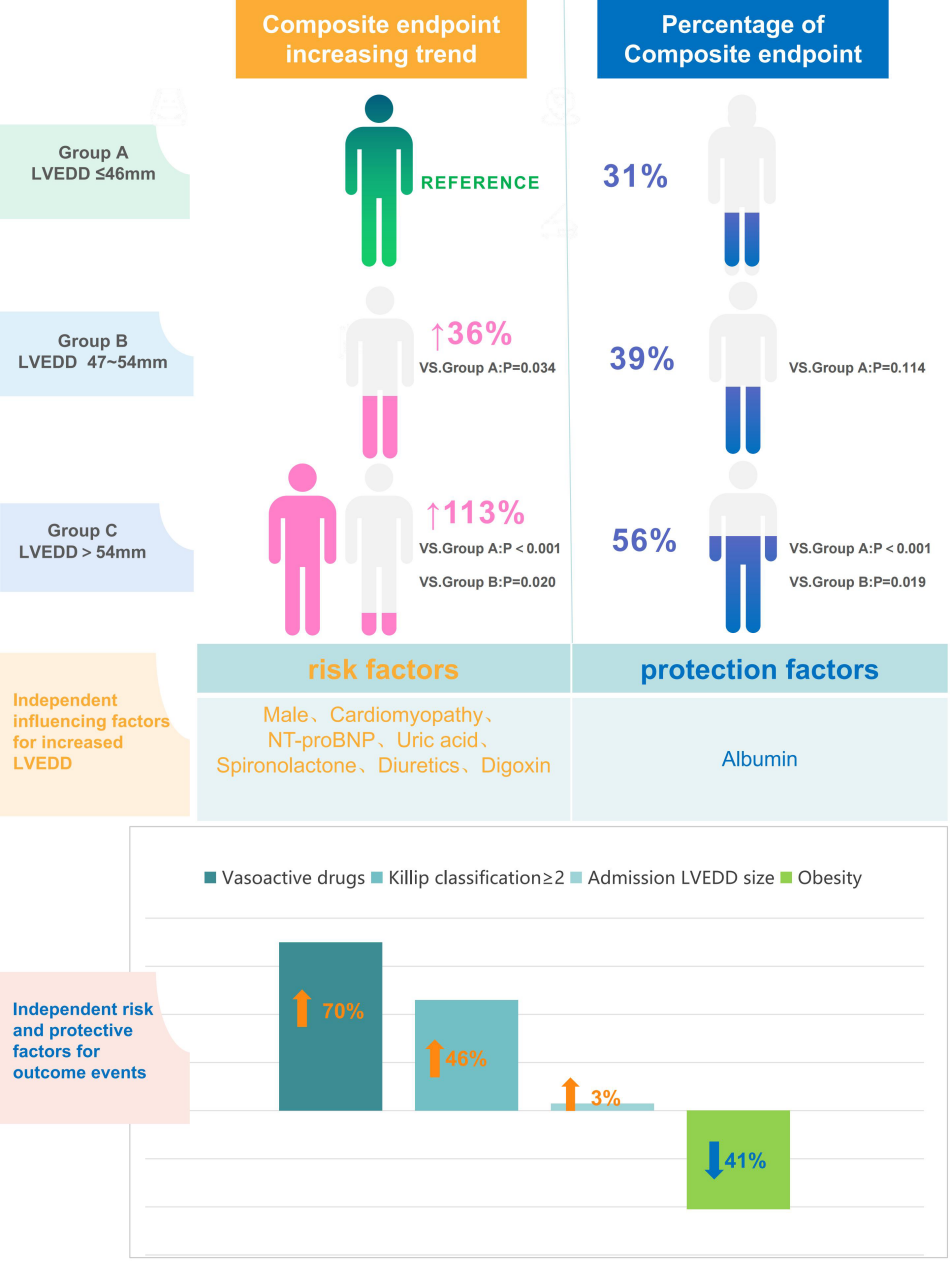
Central illustration core results.

The findings of our study demonstrate a significant association between LVEDD, measured within the first 24 h of admission, and the risk of composite endpoint events in patients with STEMI. The risk escalates substantially when LVEDD exceeds 47 mm, particularly when it surpasses 54 mm. Beyond LVEDD, we found that Killip classification ≥2, use of vasodilators, and obesity were independently associated with the risk of composite endpoint events. Additionally, we identified several independent factors associated with LVEDD size, including a history of cardiomyopathy, admission NT-proBNP, uric acid, and albumin levels, as well as the use of diuretics, spironolactone, and digoxin (Fig. 6).

Firstly, our investigation revealed a positive correlation between LVEDD and the risk of composite endpoint events, which aligns with previous findings (Van Loon et al., 2014; St John Sutton et al., 1994; Pfeffer et al., 1992). However, some studies have argued that the association between LVEDD and mortality is not statistically insignificant (Kim et al., 2017; Adelstein et al., 2017). Notably, our study differs from prior investigations as we assessed LVEDD within 24 h of admission, allowing for earlier identification of high-risk patients and the implementation of appropriate interventions.

Moreover, our research identified independent factors associated with LVEDD. For instance, a history of cardiomyopathy, elevated admission NT-proBNP and uric acid levels, and the use of spironolactone, diuretics, and digoxin were identified as factors independently associated with increased LVEDD. These associations may reflect increased cardiac load and impaired left ventricular diastolic function. Previous studies have demonstrated that left ventricular remodeling post-myocardial infarction, characterized by ventricular dilation and dysfunction, is a significant risk factor for developing clinical heart failure and can be modulated by pharmacological interventions, including ACE inhibitors and beta-blockers (St John Sutton et al., 1994; Solomon et al., 2005; Doughty et al., 2004). Our investigation explored the effects of medications and examined multiple factors related to LVEDD, offering a more comprehensive understanding. Conversely, elevated albumin levels were identified as an independent protective factor associated with LVEDD size, potentially due to the stabilizing effect of albumin on the cardiovascular system (Don & Kaysen, 2004).

Based on these findings, interventions targeting these independent risk factors may positively impact the prognosis of STEMI patients. For example, closer monitoring of cardiac function and timely treatment adjustments for patients with a history of cardiomyopathy may help mitigate the risk of LVEDD enlargement (Bolognese et al., 2002). Additionally, targeting biochemical markers such as NT-proBNP may improve prognosis in STEMI patients (Dorobantu et al., 2010).

In our study, we observed a negative correlation between LVEF and LVEDD. Univariate analysis indicated that a higher LVEF is associated with a decreased incidence of composite endpoint events. These findings align with results from Butcher et al. (2022) who reported that in STEMI patients, a lower LVEF is independently associated with an increased risk of long-term all-cause mortality (Butcher et al., 2022).

Our study also identified that Killip classification ≥ 2 and the use of vasoactive drugs were independent risk factors for composite endpoint events. This observation is consistent with the research by Vincent et al., who also identified a link between Killip classification and cardiovascular outcomes (De Geare et al., 2001). Obesity emerged as an independent protective factor for composite endpoint events, a phenomenon potentially explained by the “obesity paradox” in STEMI patients, prior studies have suggested that lower epicardial adipose tissue in heart failure patients is linked to higher mortality rates (Doesch et al., 2010). However, our study extended these findings by examining a broader range of factors affecting outcome events, enabling a more comprehensive assessment of patient prognosis.

A pivotal strength of our study was measurement of LVEDD within 24 h of admission, in contrast to previous studies that assessed LVEDD at discharge. This approach allowed for an earlier assessment of the prognostic impact of LVEDD in STEMI patients, providing clinicians with a more targeted evaluation method. Furthermore, our multiple regression analysis evaluated pre-admission risk factors, biochemical indicators, and medication usage in relation to LVEDD, identifying potential influencing factors and offering valuable insights for clinical intervention.

### Study limitations

Retrospective study design: Our study’s retrospective nature may introduce selection and information biases, as it relies on previously recorded data which may not have been collected with the current research questions in mind. For example, while we included several common cardiovascular drugs, some medications were not incorporated into the study, and variations in individual patient medication adjustments may exist.

Sample size: The relatively small sample size may affect the stability and generalizability of the results, limiting the ability to detect smaller yet clinically significant differences or associations.

Focus on STEMI patients: Our exclusive focus on STEMI patients and the exclusion of Non-ST-Elevation Myocardial Infarction (NSTEMI) patients may limit the applicability of our findings to the broader population of patients with acute coronary syndromes.

Single-center study: Being conducted in a single center, our study may reflect regional practice patterns and patient demographics, potentially limiting its generalizability to other settings or populations.

Measurement specificity and supplementary tables: The use of LVEDD, measured in the PLAX view, might not fully capture the complex three-dimensional geometry of the left ventricle. This limitation is particularly relevant in diverse STEMI scenarios such as anterior STEMI with mid LAD occlusion or RCA related inferior STEMI. Moreover, the additional prognostic factors included in Table S5 should be interpreted with caution, considering their derivation from the same dataset with inherent limitations.

Data limitations of retrospective analysis: The retrospective nature of our database may have led to incomplete data and limited availability of certain clinical variables, which could impact the study’s findings and interpretations. For example, while the database records medication information, it lacks precise timing of drug administration (relative to the ultrasound examination). Although it can be confirmed that direct PCI, antiplatelet, and anticoagulant medications were necessarily administered before the LVEDD measurement, the sequence of other adjunctive medications cannot be clearly determined. The absence of such chronological information may affect our interpretation of the causal relationship between early medication use and the initial value of LVEDD. The data in our retrospective registry were not systematically captured or coded the clinical diagnosis of STEMI location (*e.g.*, anterior, inferior) that allows for reliable statistical analysis of outcomes based on these subgroups. Given the retrospective design, body surface area (BSA) data were unavailable in our cohort, and LV dimensions could not be indexed to BSA.

Potential for confounding variables: There is a possibility that unmeasured or residual confounding variables may have influenced the results, given the complexity of STEMI pathophysiology and management.

### Clinical Implications

Despite these limitations, our findings have clinical implications. To address these limitations and build upon our results, future research could be expanded in several directions: First, larger-scale multicenter studies should be conducted to enhance the robustness and generalizability of the results. Second, the study scope should be extended to include NSTEMI patients to comprehensively evaluate the prognostic impact of LVEDD in the broader context of AMI. Third, prospective research designs to minimize the impact of selection and information bias. Finally, further exploration of other potential determinants of LVEDD and clinical outcomes—such as genetic, lifestyle, and environmental factors—is warranted to provide a more comprehensive understanding of the relationship between LVEDD and cardiovascular prognosis.

## Conclusions

In conclusion, our findings align with previous research, they offer distinct contributions by highlighting the prognostic value of early LVEDD assessment. We determined that LVEDD measured within 24 h of admission is a crucial predictor of composite endpoint events in STEMI patients. We also identified the critical LVEDD thresholds and elucidated independent risk and protective factors affecting both LVEDD size and clinical outcomes. These findings contribute to a deeper understanding of STEMI patient prognosis and offer more precise assessment methods and intervention measures for clinicians. Future research is warranted to validate these findings in larger samples and different populations to enhance their clinical applicability.

##  Supplemental Information

10.7717/peerj.21108/supp-1 Supplemental Information 1 Effect of admission LVEDD size on clinical outcome

10.7717/peerj.21108/supp-2 Supplemental Information 2 Baseline characteristics of follow-up after discharge (N=382)

10.7717/peerj.21108/supp-3 Supplemental Information 3 Effect of admission LVEDD size on mortality

10.7717/peerj.21108/supp-4 Supplemental Information 4 Effect of admission LVEDD size on cardiovascular events

10.7717/peerj.21108/supp-5 Supplemental Information 5 Other potential indicators that may significantly impact the prognosis of patients with STEMI

10.7717/peerj.21108/supp-6 Supplemental Information 6 Raw data

10.7717/peerj.21108/supp-7 Supplemental Information 7 English-language codebook

## Additional Information and Declarations

### Data Availability

The following information was supplied regarding data availability: The raw data are available in the Supplemental File.
