# Supplementary material for: Impact of left ventricular end-diastolic diameter size within 24 hours of hospital admission on outcome events in patients with ST-elevation myocardial infarction
Source: PeerJ. 2026 Apr 20;14:e21108. doi: 10.7717/peerj.21108 (PMC13105185; doi:10.7717/peerj.21108)
Supplement: Supplemental Information 1 [file peerj-14-21108-s001.docx]

| **Supplementary Table 1 Effect of admission LVEDD size on clinical outcome** | | |
| --- | --- | --- |
| **Variables** | **Hazard ratio (95%CI)** | ***P-value*** |
| Model Ⅰ | | |
| Admission LVEDD size | 1.044 (1.019, 1.070) | **<0.001** |
| Admission LVEDD size grouping |  |  |
| ≤46mm | ref. |  |
| 47~54mm | 1.356 (1.023, 1.797) | **0.034** |
| >54mm | 2.129 (1.436, 3.157) | **<0.001** |
| Model Ⅱ | | |
| Admission LVEDD size | 1.049 (1.023, 1.076) | **<0.001** |
| Admission LVEDD size grouping |  |  |
| ≤46mm | ref. |  |
| 47~54mm | 1.486 (1.108, 1.994) | **0.008** |
| >54mm | 2.279 (1.517, 3.425) | **<0.001** |
| Model Ⅲ | | |
| Admission LVEDD size | 1.051 (1.025, 1.077) | **<0.001** |
| Admission LVEDD size grouping |  |  |
| ≤46mm | ref. |  |
| 47~54mm | 1.568 (1.165, 2.110) | **0.003** |
| >54mm | 2.327 (1.545, 3.505) | **<0.001** |
| Model Ⅳ | | |
| Admission LVEDD size | 1.044 (1.018, 1.071) | **<0.001** |
| Admission LVEDD size grouping |  |  |
| ≤46mm | ref. |  |
| 47~54mm | 1.638 (1.216, 2.208) | **0.001** |
| >54mm | 2.116 (1.386, 3.231) | **<0.001** |
| Model Ⅴ | | |
| Admission LVEDD size | 1.040 (1.013, 1.067) | **0.003** |
| Admission LVEDD size grouping |  |  |
| ≤46mm | ref. |  |
| 47~54mm | 1.642 (1.217, 2.215) | **0.001** |
| >54mm | 1.961 (1.274, 3.020) | **0.002** |
| Model Ⅵ | | |
| Admission LVEDD size | 1.033 (1.006, 1.061) | **0.017** |
| Admission LVEDD size grouping |  |  |
| ≤46mm | ref. |  |
| 47~54mm | 1.601 (1.185, 2.163) | **0.002** |
| >54mm | 1.766 (1.121, 2.783) | **0.014** |
| Model ⅦI | | |
| Admission LVEDD size | 1.060 (1.030, 1.090) | **<0.0001** |
| Admission LVEDD size grouping |  |  |
| ≤46mm | ref. |  |
| 47~54mm | 1.430 (1.050, 1.940) | **0.0216** |
| >54mm | 2.540 (1.660, 3.890) | **<0.0001** |
| Hazard ratios from Cox proportional hazards regressions. Bold represent significant values (p < 0.050).  Model Ⅰ adjust for: None.  Model Ⅱ adjust for: Gender; Age.  Model Ⅲ adjust for: Gender; Age; Smoker; Drinker; Obesity.  Model Ⅳ adjust for: Gender; Age; Smoker; Drinker; Obesity; Hyperlipidemia; Hypertension; Atrial fibrillation; Diabetes mellitus; Hyperthyroidism; Stroke; Heart valve disease; Cardiomyopathy; Chronic obstructive pulmonary disease; Renal insufficiency; Anemia.  Model Ⅴ adjust for: Age; Smoker; Drinker; Obesity; Hyperlipidemia; Hypertension; Atrial fibrillation; Diabetes mellitus; Hyperthyroidism; Stroke; Heart valve disease; Cardiomyopathy; Chronic obstructive pulmonary disease; Renal insufficiency; Anemia; Killip classification.  Model Ⅵ adjust for: Age; Smoker; Drinker; Obesity; Hyperlipidemia; Hypertension; Atrial fibrillation; Diabetes mellitus; Hyperthyroidism; Stroke; Heart valve disease; Cardiomyopathy; Chronic obstructive pulmonary disease; Renal insufficiency; Anemia; Killip classification; N-terminal pro-B type natriureti peptide; Troponin T; High density lipoprotein; Creatinine; Albumin.  Model ⅦI adjust for:Model ⅦⅠ adjust for: Gender; Age; Selection of Vessel for Puncture; Major diseased vessels; Degree of vessel narrowing; Number of stents implanted; Complications; Time of presentation; Door-to-ball time.  Abbreviations:CI=conﬁdence interval;LVEDD:left ventricular end-diastolic diameter. | | |
