# Supplementary material for: Impact of left ventricular end-diastolic diameter size within 24 hours of hospital admission on outcome events in patients with ST-elevation myocardial infarction
Source: PeerJ. 2026 Apr 20;14:e21108. doi: 10.7717/peerj.21108 (PMC13105185; doi:10.7717/peerj.21108)
Supplement: Supplemental Information 2 [file peerj-14-21108-s002.docx]

| **Supplementary Table 2 Baseline characteristics of follow-up after discharge (N=382)** | |
| --- | --- |
| **Variables** | **Mean+SD / N(%)** |
| **Demographics** |  |
| Male, N (%) | 301 (78.800%) |
| Age, years | 62.601 ± 11.400 |
| Smoker, N (%) | 215 (56.301%) |
| Drinker, N (%) | 49 (12.810%) |
| Obesity, N (%) | 105 (27.510%) |
| **Medical history, N (%)** |  |
| Hyperlipidemia | 149 (39.001%) |
| Hypertension | 228 (59.701%) |
| Atrial fibrillation | 26 (6.812%) |
| Diabetes mellitus | 101 (26.403%) |
| Hyperthyroidism | 8 (2.101%) |
| Stroke | 45 (11.812%) |
| Heart valve disease | 69 (18.121%) |
| Cardiomyopathy | 21 (5.501%) |
| COPD | 52 (13.610%) |
| Renal insufficiency | 54 (14.100%) |
| Anemia | 65 (17.000%) |
| Killip classification≥2 | 140(36.612%) |
| **Serology** |  |
| N-terminal pro-B type natriureti peptide, pg/ml | 1780.500 ± 3815.600 |
| Troponin T, ng/mL | 5.100 ± 3.600 |
| Creatinine, µmol/L | 86.301 ± 83.800 |
| High density lipoprotein,mmol/L | 1.000 ± 0.300 |
| Albumin,g/L | 40.300 ± 4.000 |
| **Treatment, N (%)** |  |
| Angiotensin receptor - enkephalase inhibitors | 157 (41.100%) |
| Diuretics | 230 (60.200%) |
| Spironolactone | 90 (23.600%) |
| Vasoactive drugs | 46 (12.000%) |
| Digoxin | 6 (1.600%) |
| **Comparison of twice left ventricle sizes** |  |
| Admission LVEDD size,mm | 48.800 ± 5.200 |
| Follow-up LVEDD size,mm | 50.300 ± 5.700 |
| LVEDD size difference,mm | 1.500 ± 3.700 |
| Rate of change in LVEDD size,% | 3.200 ± 7.800 |
| **Outcome event, N (%)** |  |
| Composite endpoint | 190 (49.700%) |
