# Supplementary material for: Impact of left ventricular end-diastolic diameter size within 24 hours of hospital admission on outcome events in patients with ST-elevation myocardial infarction
Source: PeerJ. 2026 Apr 20;14:e21108. doi: 10.7717/peerj.21108 (PMC13105185; doi:10.7717/peerj.21108)
Supplement: Supplemental Information 3 [file peerj-14-21108-s003.docx]

| **Supplementary Table 3 Effect of admission LVEDD size on mortality** | | |
| --- | --- | --- |
| **Variables** | **Hazard ratio (95%CI)** | ***P-value*** |
| Model Ⅰ | | |
| Admission LVEDD size | 1.085 (1.030, 1.144) | **0.002** |
| Admission LVEDD size grouping |  |  |
| ≤46mm | ref. |  |
| 47~54mm | 1.813 (0.757, 4.340) | 0.182 |
| >54mm | 5.245 (1.996, 13.784) | **<0.001** |
| Model Ⅱ | | |
| Admission LVEDD size | 1.049 (1.023, 1.076) | **<0.001** |
| Admission LVEDD size grouping |  |  |
| ≤46mm | ref. |  |
| 47~54mm | 1.486 (1.108, 1.994) | **0.008** |
| >54mm | 2.279 (1.517, 3.425) | **<0.001** |
| Hazard ratios from Cox proportional hazards regressions. Bold represent significant values (p < 0.050).  Model Ⅰ adjust for: None.  Model II adjust for: Age; Smoker; Drinker; Obesity; Hyperlipidemia; Hypertension; Atrial fibrillation; Diabetes mellitus; Hyperthyroidism; Stroke; Heart valve disease; Cardiomyopathy; Chronic obstructive pulmonary disease; Renal insufficiency; Anemia; Killip classification; N-terminal pro-B type natriureti peptide; Troponin T; High density lipoprotein; Creatinine; Albumin.  Abbreviations:CI=conﬁdence interval;LVEDD:left ventricular end-diastolic diameter. | | |
