# Supplementary material for: Impact of left ventricular end-diastolic diameter size within 24 hours of hospital admission on outcome events in patients with ST-elevation myocardial infarction
Source: PeerJ. 2026 Apr 20;14:e21108. doi: 10.7717/peerj.21108 (PMC13105185; doi:10.7717/peerj.21108)
Supplement: Supplemental Information 4 [file peerj-14-21108-s004.docx]

| **Supplementary Table 4 Effect of admission LVEDD size on cardiovascular events** | | |
| --- | --- | --- |
| **Variables** | **Hazard ratio (95%CI)** | ***P-value*** |
| Model I | | |
| Admission LVEDD size | 1.035 (1.009, 1.062) | **0.009** |
| Admission LVEDD size grouping |  |  |
| ≤46mm | ref. |  |
| 47~54mm | 1.324 (0.984, 1.782) | **0.064** |
| >54mm | 1.772 (1.153, 2.723) | **0.009** |
| Model II | | |
| Admission LVEDD size | 1.022 (0.992, 1.054) | 0.157 |
| Admission LVEDD size grouping |  |  |
| ≤46mm | ref. |  |
| 47~54mm | 1.448 (1.054, 1.989) | **0.022** |
| >54mm | 1.428 (0.861, 2.369) | 0.168 |
| Hazard ratios from Cox proportional hazards regressions. Bold represent significant values (p < 0.050).  Model Ⅰ adjust for: None.  Model II adjust for: Age; Smoker; Drinker; Obesity; Hyperlipidemia; Hypertension; Atrial fibrillation; Diabetes mellitus; Hyperthyroidism; Stroke; Heart valve disease; Cardiomyopathy; Chronic obstructive pulmonary disease; Renal insufficiency; Anemia; Killip classification; N-terminal pro-B type natriureti peptide; Troponin T; High density lipoprotein; Creatinine; Albumin.  Abbreviations:CI=conﬁdence interval;LVEDD:left ventricular end-diastolic diameter. | | |
