# Supplementary material for: Impact of left ventricular end-diastolic diameter size within 24 hours of hospital admission on outcome events in patients with ST-elevation myocardial infarction
Source: PeerJ. 2026 Apr 20;14:e21108. doi: 10.7717/peerj.21108 (PMC13105185; doi:10.7717/peerj.21108)
Supplement: Supplemental Information 5 [file peerj-14-21108-s005.docx]

| **Supplementary Table 5 Other potential indicators that may significantly impact the prognosis of patients with STEMI.** | | | |
| --- | --- | --- | --- |
| Composite endpoint | No (N=415) | Yes (N=249) | *P-value* |
| Time of presentation, min | 288.82 ± 527.89 | 269.85 ± 422.68 | 0.63 |
| Door-to-ball time, min | 68.23 ± 25.65 | 71.76 ± 28.77 | 0.102 |
| Selection of Vessel for Puncture |  |  | 0.134 |
| Radial Artery | 391 (94.220%) | 227 (91.160%) |  |
| Femoral Artery | 24 (5.780%) | 22 (8.840%) |  |
| Major diseased vessels |  |  | 0.319 |
| Left Anterior Descending | 192 (46.270%) | 119 (47.790%) |  |
| Left Circumflex | 45 (10.840%) | 20 (8.030%) |  |
| Right coronary artery | 176 (42.410%) | 106 (42.570%) |  |
| Left main coronary artery | 2 (0.480%) | 4 (1.610%) |  |
| Degree of vessel narrowing |  |  | 0.423 |
| 90~99% | 122 (29.400%) | 66 (26.510%) |  |
| 100% | 293 (70.601%) | 183 (73.490%) |  |
| Number of stents implanted |  |  | 0.246 |
| 0 | 61 (14.701%) | 36 (14.460%) |  |
| 1 | 276 (66.511%) | 178 (71.490%) |  |
| 2 | 67 (16.141%) | 33 (13.250%) |  |
| 3 | 11 (2.651%) | 2 (0.800%) |  |
| Complications |  |  | 0.003 |
| none | 310 (74.701%) | 170 (68.271%) |  |
| shock | 22 (5.301%) | 18 (7.231%) |  |
| mechanical complications | 1 (0.241%) | 5 (2.011%) |  |
| stroke | 3 (0.721%) | 2 (0.801%) |  |
| bleeding | 7 (1.691%) | 6 (2.411%) |  |
| infection | 68 (16.391%) | 35 (14.061%) |  |
| coronary artery thrombosis formation | 1 (0.241%) | 1 (0.401%) |  |
| respiratory failure | 2 (0.481%) | 1 (0.401%) |  |
| recurrent myocardial infarction | 1 (0.241%) | 1 (0.401%) |  |
| in-hospital death | 0 (0.000%) | 10 (4.021%) |  |
